# THE PARADOX OF PAID CARE: BENEFITS AND CHALLENGES FOR SPOUSAL CAREGIVERS’ EMOTIONAL WELL-BEING

**DOI:** 10.1093/geroni/igae098.3386

**Published:** 2024-12-31

**Authors:** Leslie Fontaine, Jyoti Savla, Karen Roberto

**Affiliations:** Virginia Tech, Blacksburg, Virginia, United States; Virginia Tech, Blacksburg, Virginia, United States; Virginia Tech, Blacksburg, Virginia, United States

## Abstract

Recent research suggests that social factors may buffer negative outcomes of caregiving, including social isolation. In particular, spouse caregivers of partners living with moderate to severe dementia (PLwD) often find themselves confined to their homes, which reduces their opportunities for social interactions and intensifies feelings of loneliness and caregiver stress and burden. For many spouse caregivers, their primary in-person interactions are with paid caregivers. The aim of this study was to investigate if the use of paid helpers mitigated feelings of loneliness and overload among caregivers. Fifty-one rural spouse caregivers (61% wives, 98% White, 87% residing only with PLwD), answered structured interview questions that assessed their levels of loneliness, role overload, and degree of assistance from paid helpers. Low levels of overload and loneliness was experienced by 16% spouses, with 88% of them not utilizing paid services. In contrast, 65% of spouses reported significant levels of both overload and loneliness. Within this group, 62% of wives used paid helpers, whereas 75% of husbands did not. Multinomial regressions indicated that husbands without paid helpers experienced more pronounced feelings of loneliness and overload, whereas wives with paid helpers reported increased feelings of loneliness and overload, suggesting that paid support does not uniformly alleviate the emotional strain and loneliness of caregiving. Findings underscore the complex gender differences associated with paid support on the emotional well-being of spouse caregivers. Discussion highlights the benefits of paid assistance in addressing multifaceted caregiving outcomes and the need for more tailored and nuanced support strategies for spouse caregivers.

